# Quzhuo tongbi formula for reducing gout flare related to uric acid lowering treatment: Study protocol for a multiple-center, randomized, double-blind, placebo, parallel-controlled clinical trial

**DOI:** 10.1371/journal.pone.0327864

**Published:** 2025-07-09

**Authors:** Hejing Pan, Xuanlin Li, Shan Wu, Donghai Zhou, Haojie Guan, Haoyu Wu, Qiao Wang, Yujun Tang, Xuanming Hu, Meijiao Wang, Mingqian Zhou, Runyue Huang, Lei Liu, Yaolong Chen, Chengping Wen, Lin Huang

**Affiliations:** 1 College of Basic Medical Science, Zhejiang Chinese Medical University, Hangzhou, China; 2 the First Affiliated Hospital of Zhejiang Chinese Medical University, Hangzhou, China; 3 the Second Affiliated Hospital of Zhejiang Chinese Medical University, Hangzhou, China; 4 Shengzhou People’s Hospital, Shengzhou, China; 5 Huzhou Hospital of Traditional Chinese Medicine, Huzhou, China; 6 the Second Affiliated Hospital of Guangzhou University of Chinese Medicine,; 7 Department of Rheumatology and Immunology, the Second Affiliated Hospital of Zhejiang University, Hangzhou, China; 8 Center for Evidence-Based Medicine, Lanzhou University, Lanzhou, China; University of Illinois, UNITED STATES OF AMERICA

## Abstract

**Introduction:**

Gout, a prevalent metabolic rheumatic disease, arises from the accumulation of monosodium urate (MSU) crystals in joints, directly associated with hyperuricemia. Long-term or lifelong urate-lowering therapy (ULT) stands as the primary management strategy. However, there is a significant risk of gout flares during ULT, and current methods have drawbacks, including side effects. Previous research has suggested a potential benefit of the Qu-zhuo Tong-bi formula (QTF) in the treatment and prevention of gout. This study aims to evaluate the effectiveness and safety of QTF in reducing gout flares during ULT.

**Method:**

This is a multi-center, randomized, double-blind, placebo, parallel-controlled clinical trial conducted in four hospitals in Zhejiang Province, China. A total of 144 patients, aged 18–80 years, will be randomly allocated to either the intervention group (QTF + febuxostat) or the control group (placebo + febuxostat) for a 12-week treatment period. The primary outcome measure is the frequency of gout flares. Secondary outcome measures include the time from the patient’s enrollment to the first occurrence of gout flare (in days), duration of gout flare (in days), health assessment questionnaire disability index (HAQ-DI), pain level (visual analog scale – VAS), rate of achieving target uric acid levels. Occurrences of adverse events, and results from electrocardiograms and laboratory tests, will be monitored.

**Discussion:**

The findings from this study will offer valuable evidence supporting the efficacy of the QTF in reducing gout flares during ULT.

**Trial registration:**

ChiCTR20084417 Register on May 16, 2024.

## Introduction

Gout is a common metabolic rheumatic disease, and the incidence of affected individuals is increasing [1]. It is characterized by the deposition of monosodium urate (MSU) crystals in the joints, with elevated serum urate concentration (hyperuricemia) being the primary risk factor. Long-term or lifelong urate-lowering therapy can dissolve MSU crystals and reduce the body’s urate level, ultimately alleviating gout flares, making it a fundamental approach in gout management [2]. The initiation of urate-lowering therapy (ULT) is known to carry a risk of gout flares [3,4]. Studies have shown that within the first 3–6 months of starting ULT, the recurrence rate of gout flares ranges from 15% to 70% in patients with hyperuricemia and gout [5–7], potentially increasing the risk of cardiovascular events [8]. Moreover, gout, being a recurrent chronic ailment, not only causes physical discomfort for patients but also imposes a substantial economic burden [9]. Therefore, reducing gout flares is crucial when initiating ULT.

Currently, clinical practice guidelines recommend medications such as colchicine, non-steroidal anti-inflammatory drugs (NSAIDs), or corticosteroids for the management of gout flares during ULT [10]. However, colchicine is associated with significant gastrointestinal, hepatic, and renal toxicity and may potentially suppress bone marrow, necessitating caution [11–13]. Prolonged use of NSAIDs and corticosteroids can lead to serious complications, including gastrointestinal ulcers, which may impact patient adherence to ULT. Given the potential side effects and limitations of current medication for gout prevention and treatment, there is an urgent need to explore new approaches to reduce gout flares.

Several approaches have been explored to address gout flares associated with fluctuations in serum urate levels. Canakinumab, administered in doses ranging from 50 to 300 mg as a single dose or through a 4-weekly dosing regimen over 4 months, has shown effectiveness in preventing gout flares during ULT, surpassing the efficacy of colchicine alone and demonstrating good tolerability. However, it’s worth noting that this study excluded patients with potential risks related to either treatment, which may limit the generalizability of the findings to the broader population [14]. Studies have shown that rilonacept significantly decreases the frequency of gout flares during initial ULT treatment, with a favorable safety profile. While the incidence of infections with rilonacept treatment did not exceed that of the placebo in this relatively small-scale study, there remains a possibility, as observed with other IL-1 inhibitors, of an elevated risk for specific infections [15]. Currently, some studies suggest that adopting a low-dose initiation, stepwise dose-increased ULT regimen can reduce gout flares, potentially substituting for medication-based preventative treatment strategies [16–18]. However, the open-label nature of these studies could have influenced the results [16,17]. One study may have failed to achieve its primary endpoint due to an inadequate sample size [17], while another study’s protocol required the exclusion of individuals with stage 4 or 5 chronic kidney disease, limiting the generalizability of its findings, particularly in terms of safety analysis [18]. These approaches offer enhanced efficacy and safety, compared to traditional methods, providing valuable insights for clinical treatment. Nonetheless, the inherent adverse reactions of pharmacological treatments, in conjunction with factors influencing the certainty and applicability of results, underscore the importance of exploring innovative strategies and treatment modalities.

In recent years, Traditional Chinese Medicine (TCM) has been increasingly recognized for its effectiveness [19]. When combined with chemical drugs, TCM has shown enhanced efficacy in halting disease progression compared to chemical drugs alone [20]. Research has indicated that the aqueous extract of Tongfengkang not only possesses a significant anti-inflammatory effect on gouty arthritis (GA) but also lowers blood urate concentration [21]. Previous studies conducted by our team have demonstrated that Qu-zhuo Tong-bi formula (QTF) granules are effective in reducing blood urate levels and acute gout flares, with no adverse reactions reported even after prolonged use. However, these studies did not include follow-up after treatment [22]. Despite these promising results, there remains a gap in traditional Chinese medicine (TCM) research concerning the prevention of gout flares during urate-lowering therapy (ULT). To address this gap, we propose a multi-center, randomized, double-blind, placebo-controlled, parallel-group clinical trial. This study aims to investigate whether QTF granules can reduce the frequency and duration of acute gout flares associated with ULT and prolong the time to flare. Additionally, we will address the existing research gap by including a 48-week follow-up period after treatment to evaluate the long-term effects of QTF granules. The aim is to identify a safe and effective new therapeutic medication for reducing acute gout flares in the early stages of ULT in gout patients, providing valuable insights and a foundation for TCM clinical management and novel drug development.

## Methods

### Study design

A multi-center, randomized, double-blind, placebo, parallel-controlled clinical trial will be conducted in four hospitals across the province of Zhejiang, including the First Affiliated Hospital of Zhejiang Chinese Medical University, the Second Affiliated Hospital of Zhejiang Chinese Medical University, Shengzhou People’s Hospital, and Huzhou Hospital of TCM.

**Fig 1:**
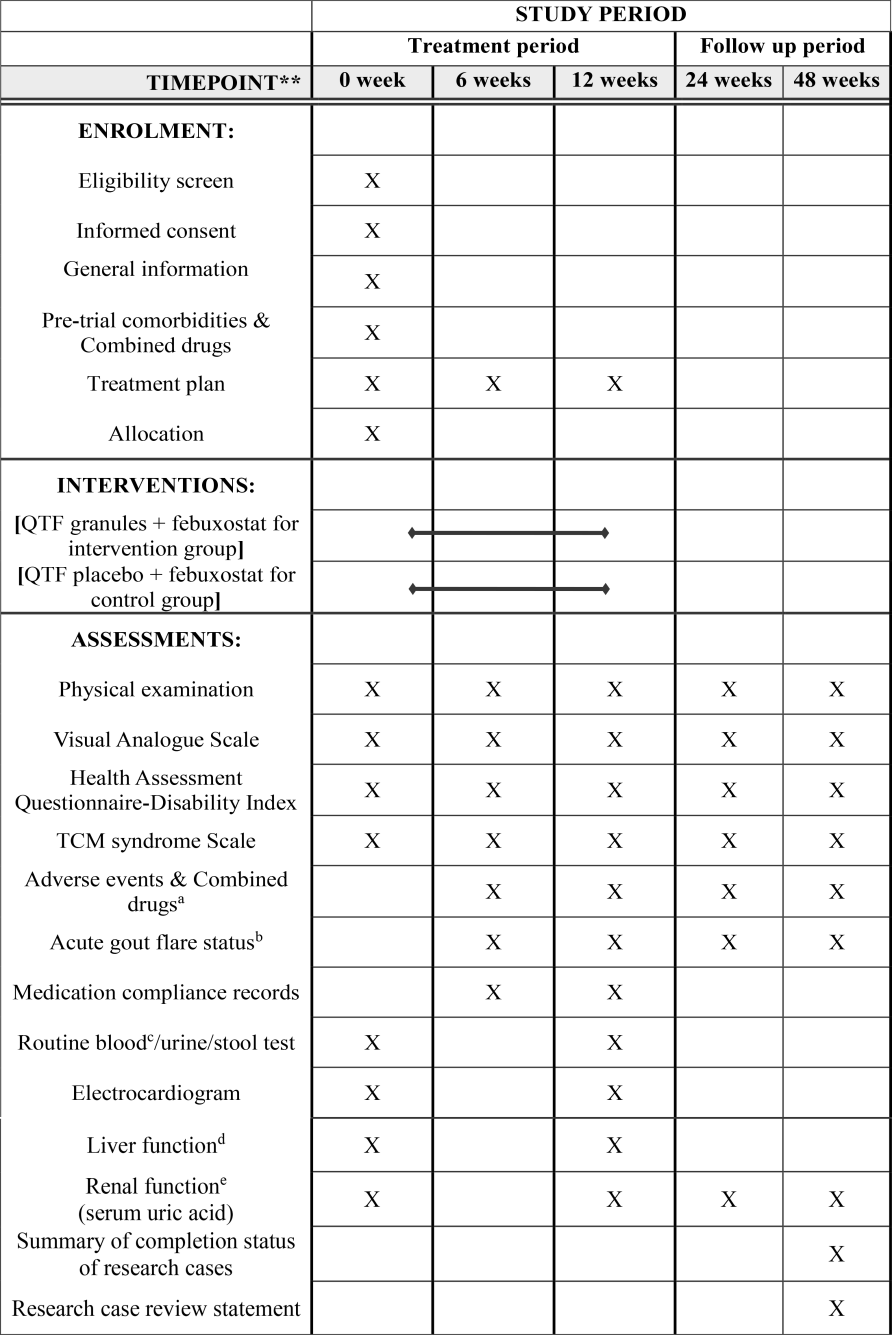
Study procedure. ^a^Observe continuously throughout the study and record when it occurs. ^b^Including the number of acute flares of gouty arthritis, the time span from randomization to the first occurrence of an acute attack, and the duration of recurrent acute gouty arthritis. ^c^Red blood cell count (RBC), white blood cell count (WBC), hemoglobin (HGB), neutrophil percentage (NEU), platelet count (PLT). ^d^Alanine aminotransferase (ALT), aspartate aminotransferase (AST). ^e^Blood urea nitrogen (BUN), creatinine (Cr), serum uric acid (SUA).Qu-zhuo Tong-bi formula(QTF).

**Fig 2:**
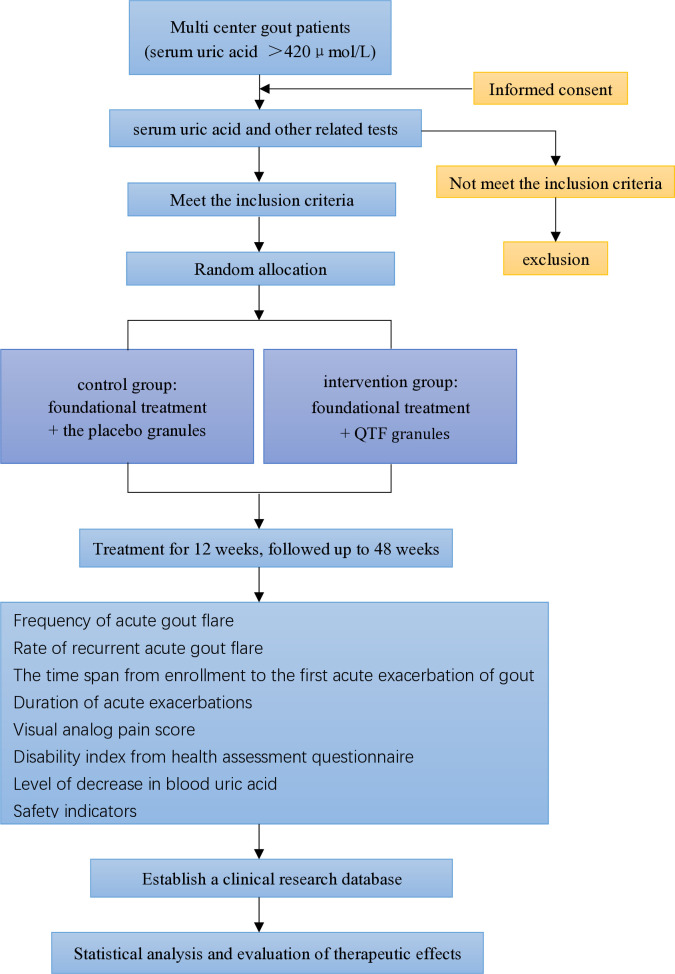
The flow diagram of the study.

This protocol adheres to the SPIRIT 2013 reporting checklist prohibit [23]. The study will also proceed according to the Declaration of Helsinki (as revised in 2013). The entire study flowchart and follow-up procedures are illustrated in Fig 1 and Fig 2, respectively.

### Participants

#### Recruitment.

The participant recruitment process involves primarily several key steps, including recruitment screening, and obtaining informed consent from participants. Blood, urine, and stool samples will be collected with participants’ consent for further research. The recruitment process includes the following steps: 1. Developing a recruitment plan and determining needs, advertising through various channels such as recruitment advertisements in the outpatient hall, entrances of the wards, and announcements on WeChat (a popular social platform in China). 2. Assigning doctors from each center to recruit individuals and having dedicated staff to assist participants. Participants are selected based on criteria and doctors’ clinical experience. 3. Providing detailed descriptions of the clinical trial protocol to qualified participants to ensure an understanding of potential benefits and risks.

### Patients’ eligibility and criteria

We will recruit a total of 144 participants aged 18–80. The diagnostic criteria were as follows:

1. Diagnosis of gout: Referring to the 2015 ACR/EULAR classification criteria for gout, with a maximum score of 23 points, a diagnosis of gout can be made when the score is ≥ 8 points [24].
2. The TCM diagnosis of spleen deficiency dampness obstruction syndrome: T/CACM1349–2021- Group standards of China Association of Chinese Medicine. (available in Table 1)

**Table 1:** Differential diagnosis criteria for syndrome of spleen deficiency with dampness obstruction.

Primary symptoms and signs	1. Difficulty in joint flexion and extension
	2. Joint wandering pain
	3. > 1 joint pain
	4. feeling of fullness and oppression in the epigastrium.
Secondary symptoms and signs	1. Heaviness in the limbs
	2. Abnormal bowel movements (loose/stool/alternating between loose and hard/initially hard then loose)
	3. Decreased appetite
	4. Pale tongue
	5. Soft or thin pulse

Meeting at least 4 primary symptoms and signs or 3 primary symptoms and signs along with 2 secondary symptoms and signs would indicate a diagnosis of spleen deficiency with dampness obstruction.

### Inclusion criteria

All participants must meet the following criteria:

1. Meet the above diagnostic criteria and be in the gout intermission stage;
2. TCM diagnosis indicates syndrome of spleen deficiency with dampness obstruction;
3. Serum urate level ≥7 mg/dL (420μmol/L);
4. Aged between 18 and 80 years (inclusive of boundary values), any gender;
5. Having not received urate-lowering drugs such as allopurinol, benzbromarone, or febuxostat in the past month;
6. Relief of gouty flare symptoms (VAS score = 0) for more than 1 week;
7. Acute gout flare≥2 in the past year;
8. Willing to undergo treatment and sign informed consent.

### Exclusion criteria

Patients meeting any of the following criteria will be excluded:

1. Severe liver or kidney dysfunction (AST/ALT ≥ twice the normal level, eGFR < 60ml/min), diabetes with target organ damage, severe cardiovascular or cerebrovascular diseases, history of digestive ulcer bleeding or perforation, and patients with psychiatric disorders;
2. Patients’ intolerance to or with contraindications to the medications used in this study (urate-lowering medications, traditional Chinese medicine, nonsteroidal anti-inflammatory medications);
3. Patients with hyperuricemia caused by myeloproliferative disorders or other conditions with obvious urate overproduction;
4. Pregnant or lactating patients.

### Exit criteria

#### 1. Investigator-initiated exit.

Exiting the trial refers to situations where enrolled subjects encounter circumstances during the trial that make it inappropriate for them to continue participating, as determined by the investigator. These circumstances may include 1) Poor subject compliance (< 80%), concurrent use of medications outside the protocol, or discontinuation of treatment measures midway through the trial by the subject; 2) Poor patient compliance (medication compliance <80% or >120%), self-initiated medication changes, or additions of medications concomitant medicationed by the protocol; 3) Unblinding midway due to various reasons; 4) Occurrence of certain complications, adverse events (AEs), or special physiological changes during the trial, judged by the physician as unsuitable for continued treatment, leading to withdrawal; 5) Allergic reactions or severe AEs occurring during the trial, leading to trial termination based on physician judgment.

#### 2. Subject-requested exit.

1) Patients who are unwilling or unable to continue with the clinical trial may request withdrawal from the trial from the principal investigator, leading to trial discontinuation. This includes cases where the patient cannot complete the entire course of treatment, leading to incomplete data collection affecting efficacy and safety assessments; 2) Subjects who, although not explicitly requesting withdrawal from the trial, no longer accept medication or testing and become lost to follow-up.

### Sample size

The sample size calculation was performed using PASS (version 2021) with the “Comparison of two independent sample rates” calculation module. A power of 80% and an alpha level of 0.025 for a one-sided test and a superiority test were used. Based on a previous study by our team [22], the flare rates of acute gouty arthritis after 12 weeks of placebo treatment were 63.2%, and for the group treated with the QTF was 38.5%. Calculations yielded sample sizes of N1 = N2 = 61 for both the intervention and control groups, considering a dropout rate of 20%. In total, 144 patients with intermittent gout will be included, with 72 patients in both the intervention and control groups.

### Ethics, oversight, and dissemination

This trial has received approval from the Ethics Committee of Zhejiang Chinese Medical University, with approval number 20240329−6. All participating centers have obtained approval from their respective local institutional review boards. The Ethics Committee of Zhejiang Chinese Medical University will oversee the entire process of the trial, including assessing the status and quality of the research, such as recruitment rates, dropout rates, and AEs. Researchers will promptly address any AEs reported by patients or their families. The results of the RCT will be published in peer-reviewed journals, ensuring patient anonymity by not disclosing their names unless required by law. Access to patient information will be restricted to authorized individuals who will strictly manage data according to government and legal regulations.

### Randomization and blinding

A multi-center competitive stratified block randomization method was utilized. Random numbers for the multi-center group were generated using the corresponding randomization procedure in SAS software (version 9.4). These random numbers, alongside their corresponding drug codes, functioned as unique identifiers for participants. After conducting necessary baseline assessments, all eligible patients who met the inclusion criteria and signed informed consent forms will be randomly assigned to either the intervention or control group in a 1:1 ratio. The randomization design and numbers were provided by Jiangsu Famous Medicine Co., Ltd. The relevant parameters and results of the randomization scheme will be recorded by Zhejiang Chinese Medical University (the research design unit). Apart from the outcome evaluators and staff at the drug production center, all researchers and patients remained unaware of treatment allocation. The QTF granules and placebo granules were identical in odor, color, and packaging, ensuring blinding. Distribution details will be disclosed to researchers only when necessary for drug administration. Products were delivered directly to patients according to their allocation codes. In emergencies, the research design unit will promptly provide researchers with randomized numbers and treatment allocations. All participants will strictly adhere to confidentiality regulations to protect patients’ personal privacy information.

### Intervention

The visit schedule is set for weeks 0, 6, 12, 24, and 48. Visits at weeks 0, 6,12, and 24 will be conducted offline, with patients attending the hospital to have relevant information collected by the responsible physician. The visit at week 48 will be conducted online (via phone calls or WeChat).

Conventional treatment: Both groups receive ULT, which in this study is stipulated as febuxostat 40 mg once daily.

TCM Treatment: The intervention group receives QTF granules, while the control group is administered placebo granules, with treatment lasting for 12 weeks. QTF granules constitute a Chinese herbal compound, and the composition of the granules is detailed in Table 2. The placebo granules utilized in this study contain 10% of the same components found in QTF granules. All QTF granules and placebo granules are manufactured and packaged by Jiangyin Tianjiang Pharmaceutical Co., Ltd., located in Jiangsu Province, China. There are no discernible differences in appearance, color, odor, or weight between the QTF granules and placebo granules. The quality of these granules underwent inspection by authorized agencies before declaration, ensuring compliance with specified quality standards. Each dose of QTF granules or placebo granules consists of 9.8g per sachet, to be taken twice daily with 200 ml of warm water, continuously for 5 days followed by a 2-day rest period.

**Table 2:** Components of Qu-zhuo Tong-bi formula granules.

Chinese name	Latin name	Parts of the substances	Amount (g)
Tu Fu Ling	*Smilacis Glabrae Rhizoma*	Root and rhizome	60
Bi Xie	*Dioscoreae Hypoglaucae Rhizoma*	Root and rhizome	30
Yu Mi Xu	*Stigma Maydis*	Style and stigma	15
Chao Yi Yi Ren	*Coicis Semen*	Mature seed	30
Xi Xian Cao	*Siegesbeckiae Herba*	Aboveground parts	18
Jiang Huang	*Wenyujin Rhizoma Concisum*	Root and rhizome	12
Sang Ji Sheng	*Taxilli Herba*	stem or branches with leaves	15
Yan Hu Suo	*Corydalis Rhizoma*	Root tuber	18
Fo Shou	*Citri sarcodactylis Fructus*	Mature fruit	12

During the treatment period, in the event of gout flares, patients are advised to rest in bed with the affected limb elevated. Within 24 hours, they are required to contact the research personnel for a Visual Analog Scale (VAS) pain assessment: mild (VAS score < 5), moderate (5 < VAS score < 8), severe (VAS score > 8). Early administration of an adequate dose of NSAIDs is recommended within 24 hours. In this study, it is specified as a once-daily administration of 120 mg of etoricoxib (with an onset of action within 1 hour and a half-life of approximately 22 hours), with dosage reduction upon relief of symptoms. Blood and urine samples are collected from patients who consent for further research purposes. In the case of AEs, the use of QTF granules or placebo granules is temporarily suspended until the condition stabilizes.

The observing physician will meticulously record the quantity of medication received, used, and returned by the participants to assess medication adherence and ensure timely documentation in the case report forms. Based on the participant’s adherence, a decision will be made regarding their continued participation in the trial. The exclusion criteria for poor adherence are medication adherence rate = (actual medication amount/ theoretical medication amount) < 80% or > 120%. The medication administrator is responsible for locking the cabinet, sealing the medications, and storing them at room temperature. If a participant withdraws from the study early, any remaining medication should be collected and returned.

Regulations on Concurrent Treatments: During the trial, the use of various anti-rheumatic drugs, muscle relaxants, and analgesics, including both traditional Chinese and Western medicines such as Shenling Baizhu Powder/ Pills and Pingwei Powder, is generally prohibited. Additionally, physical therapies such as massage and acupuncture are not allowed.

If participants need to continue taking other medications or undergoing other treatments due to concurrent diseases, these must be documented in detail in the concurrent medication log.

### Outcomes

#### Primary outcomes.

The frequency of gout flares will be recorded at weeks 6, 12, 24, and 48.

#### Secondary outcomes.

1. The Time span from patient’s enrollment to the first occurrence of gout flares (in days); 2. Duration of gout flares (in days); 3. Health Assessment Questionnaire Disability Index (HAQ-DI) for quality-of-life assessment; 4. Changes in visual analog scale (VAS). 5. Rate of achieving target uric acid levels (Compliance rate = number of eligible patients/total number of patients under observation x 100%; Standard: Gout treatment achievement is defined as lowering serum uric acid levels to below 360 umol/L through the use of uric acid-lowering medications). Outcomes 1–4 will be recorded at weeks 6, 12, 24, and 48. Outcome 5 will be recorded at weeks 12, 24, and 48.

#### Safety outcomes.

Patients undergo pre- and post-treatment examinations, including blood routine, urine routine, stool routine, liver and kidney function tests (ALT, AST, BUN, Cr, SUA), and electrocardiography, to assess the safety of the interventions in this study. The blood, urine, and stool specimens collected from the patients after completion of the tests are to be disposed of by medical staff according to regulations.

We employ the Common Terminology Criteria for Adverse Events (CTCAE) version 5.0 from the National Cancer Institute of the United States to document the severity of adverse events, categorized from grades 1–5 [25]. According to the classification system devised by the Drug Adverse Reaction Monitoring Center of the Chinese Ministry of Health, adverse events are classified into five levels: “definitely related, probably related, possibly related, probably unrelated, definitely unrelated.” The first three levels are deemed to be associated with the investigational medication [26]. Any adverse events during treatment will be documented based on severity and their causal assessment outcomes. In the event of serious adverse events during the study, immediate measures must be taken and reported to the research design unit’s project manager and ethics committee, which will determine whether to proceed or terminate the study. All adverse events should be monitored until appropriately resolved or stabilized.

### Data management, monitoring and auditing

Data for this trial will be collected from Case Report Forms (CRFs) at each center, with study monitors reviewing the forms to ensure completeness and quality. Two independent data managers will be responsible for entering the data into the system. Researchers will strictly maintain participant confidentiality, with access granted only by permission from the research design unit. The data management system is provided by Jiangsu Famous Medicine Co., Ltd.

The Data Monitoring Committee (DMC), organized and led by Zhejiang Chinese Medical University, comprises experienced clinical researchers and esteemed academic scholars in the field. The committee will conduct regular audits of the data to ensure the integrity and safety of the study. These audits will adhere to internationally recognized standards for clinical trial inspections, including the examination of clinical trial items, quality, and risk management, to ensure the authenticity and integrity of the research data. Once recruitment reaches 50%, the committee will perform interim analyses of the primary outcomes using Lan-DeMets with O’Brien-Fleming methodology to evaluate the study’s effectiveness and safety.

### Statistical analysis

The statistical analysis for this trial will be conducted based on the Per-Protocol (PP) and Intent-to-Treat (ITT) data, conducted by Jiangsu Famous Medicine Co., Ltd., situated in Nanjing, China. Different analysis methods will be employed depending on the type of data measurement or counting.

For the primary outcome, the number of gout flares in each group will be compared using t-tests or Wilcoxon rank-sum tests, with center effects controlled through covariance analysis. For count data, such as time span to the first occurrence of gout flares, duration of acute gouty arthritis recurrence, pain visual analog scale score, HAQ-DI, and safety analysis indicators (like routine blood tests), the same methods will be applied. Measurement data, including the recurrence rate of acute gouty arthritis and the incidence of adverse events, will be analyzed using chi-square tests or Fisher’s exact tests, with center effects controlled through binary or multinomial logistic regression analysis. All hypothesis tests in this study will be two-sided, with a significance level of 0.05. Differences will be considered statistically significant when p < 0.05. SAS version 9.4 will be used for all statistical analyses.

## Discussion

The consensus that ULT can potentially trigger gout is well-established, with increasing evidence suggesting that prophylactic measures during ULT can reduce the recurrence rate of gout [17,27,18].

Based on the core pathogenesis and treatment principles of gout, we have formulated QTF, which is widely used in clinical practice with excellent efficacy. Previous studies have shown that QTF can effectively lower the blood uric acid levels of gout patients during the intermission stage, including the chronic period, prevents the recurrence of gout flares, and has good safety profiles [22]. Our previous study also revealed the mechanism by which QTF treats gouty arthritis. It can enhance the abundance of butyrate-producing bacteria and the production of short-chain fatty acids (SCFAs), restore the intestinal barrier, and regulate the differentiation of CD4 T cells through the PI3K-AKT-mTOR pathway, thereby suppressing the production of intestinal inflammatory factors [28,29]. Additionally, we closely monitor patients’ health-related assessments, such as HAQ-DI. Common medications used to manage gout typically include colchicine, NSAIDs, and corticosteroids. Colchicine, in particular, is recommended as a primary preventive medication by esteemed bodies such as the British Society for Rheumatology (BSR) and the European League Against Rheumatism (EULAR) [18]. It’s noteworthy that up to 90% of individuals with gout may exhibit contraindications to NSAIDs, including conditions such as hypertension, cardiovascular disease, chronic kidney disease, and gastrointestinal disorders. Similarly, as many as 40% of gout patients may have relative contraindications to colchicine, such as chronic kidney disease and chronic hepatitis [15]. In contrast, TCM offers a relatively safe alternative that can effectively address these limitations. Our team is currently seeking approval for an exploratory clinical trial aimed at assessing the safety and feasibility of QTF granules in reducing gout flare during ULT. The findings derived from this study will furnish valuable evidence to inform the future development of high-quality, large-scale randomized controlled trials.

This study has several limitations. Firstly, our research is focused on Zhejiang Province and has not been extended nationwide, resulting in a relatively homogeneous source of participants. Secondly, the dosage of febuxostat prescribed in our study is 40 mg/day, which is the typical dosage for reducing serum uric acid to below 6.0 mg/dL (356.91µmol/L) in East Asian populations (Japan and China). However, in the USA or Europe, a dosage of 80 mg/day is required to achieve the same uric acid-lowering effect [17,30,31]. This discrepancy may be attributed to differences in ethnicity and lifestyle environments, thus limiting the generalizability of the results of this study to gout patients in the USA or Europe. Another constraint is the relatively limited funding, resulting in a small estimated sample size and short follow-up duration for this exploratory study. However, based on the results obtained from this study, a future long-term, large-sample clinical trial can be conducted.

## Supporting information

S1 Data SPIRIT 2013-checklist. (DOC)

## Abbreviations

MSU: Monosodium urate
ULT: Urate-lowering therapy
QTF: Qu-zhuo Tong-bi formula
NSAIDs: Non-steroidal anti-inflammatory drugs
TCM: Traditional Chinese Medicine
GA: Gouty arthritis
RBC: Red blood cell count
WBC: White blood cell count
HGB: Hemoglobin
NEU: Neutrophil percentage
PLT: Platelet count
ALT: Alanine aminotransferase
AST: Aspartate aminotransferase
BUN: Blood urea nitrogen
Cr: Creatinine
SUA: Serum uric acid
VAS: Visual Analog Scale
AEs: Adverse events
HAQ-DI: Health Assessment Questionnaire Disability Index
CTCAE: Common Terminology Criteria for Adverse Events
CRFs: Case Report Forms
DMC: Data Monitoring Committee
PP: Per-Protocol
ITT: Intent-to-Treat
SCFAs: Short-chain fatty acids
BSR: British Society for Rheumatology
EULAR: European League Against Rheumatism
