# Correction: Quzhuo tongbi formula for reducing gout flare related to uric acid lowering treatment: Study protocol for a multiple-center, randomized, double-blind, placebo, parallel-controlled clinical trial

**DOI:** 10.1371/journal.pone.0355310

**Published:** 2026-08-03

**Authors:** Hejing Pan, Xuanlin Li, Shan Wu, Donghai Zhou, Haojie Guan, Haoyu Wu, Qiao Wang, Yujun Tang, Xuanming Hu, Meijiao Wang, Mingqian Zhou, Runyue Huang, Lei Liu, Yaolong Chen, Chengping Wen, Lin Huang

The Funding statement for this article is incorrect. The correct Funding statement is as follows: This study was supported by the Department of Science and Technology of the State Administration of Traditional Chinese Medicine-Zhejiang Province Joint Construction Project (GZY-ZJ-KJ-24012).
